# Current clinical practice for thromboprophylaxis management in patients with Cushing’s syndrome across reference centers of the European Reference Network on Rare Endocrine Conditions (Endo-ERN)

**DOI:** 10.1186/s13023-022-02320-x

**Published:** 2022-05-03

**Authors:** F. M. van Haalen, M. Kaya, I. C. M. Pelsma, O. M. Dekkers, N. R. Biermasz, S. C. Cannegieter, M. V. Huisman, B. J. M. van Vlijmen, R. A. Feelders, F. A. Klok, A. M. Pereira, Endo-ERN Cushing and Thrombosis study group, K. Stochholm, E. Fliers, F. Castinetti, T. Brue, J. Bertherat, C. Scaroni, A. Colao, R. Giordano, M. R. Druce, A. Beckers, J. Spranger, N. Driessens, D. Maiter, U. Feldt-Rasmussen, R. Feelders, S. M. Webb, M. Dattani, E. Husebye, B. Zilaitiene, S. Gaztambide, F. Gatto, D. Ferone, L. Persani, I. Chiodini, C. Höybye, A. M. Pereira, N. R. Biermasz, F. A. Klok, O. M. Dekkers, O. C. Meijer, M. Reincke, G. Vila, C. Perry, A. Heck, M. R. Stancampiano, A. van de Ven, G. Johannsson, O. Ragnarsson, M. Tóth, V. Volke, M. Toumba, L. Canu, J. Vojtková, M. Al-Mrayat, M. Fassnacht, M. Detomas, N. Karavitaki, M. M. van der Klauw, U. Groselj, A. Elenkova, D. Unuane

**Affiliations:** 1 Department of Medicine, Division of Endocrinology, Leiden University Medical Center, Leiden, Netherlands; 2 Division of Thrombosis and Hemostasis, Leiden University Medical Center, Leiden, Netherlands; 3 Department of Clinical Epidemiology, Leiden University Medical Center, Leiden, Netherlands; 4 Department of Medicine, Division of Endocrinology, Erasmus University Medical Center, Rotterdam, Netherlands; 5 Department of Endocrinology, Aarhus University Hospital, Århus, Denmark; 6 Department of Endocrinology and Metabolism, Amsterdam University Medical Centers, Location AMC, Amsterdam, Netherlands; 7 Department of Endocrinology, Assistance Publique-Hôpitaux de Marseille, Marseille, France; 8 Department of Endocrinology; Assistance Publique -Consortium Cochin, Robert Debré, Necker, St Antoine, La Pitié Salpétrière, Paris, France; 9 Endocrinology Unit, Dep Medicine, DIMED, Hospital-University of Padova, Padua, Italy; 10 Department of Endocrinology, Azienda Ospedaliera Universitaria “Federico II”, Napoli, Italy; 11 Department of Endocrinology, Azienda Ospedaliero Universitaria Città della Salute e della Scienza di Torino, Turin, Italy; 12 Department of Endocrinology, Barts Health -NHS Foundation Trust, London, UK; 13 Department of Endocrinology, Centre Hospitalier Universitaire de Liège, Liège, Belgium; 14 Department of Endocrinology and Metabolic Diseases, Charité Universitätsmedizin, Berlin, Germany; 15 Department of Endocrinology, Cliniques Universitaires de Bruxelles -Hôpital Erasme, Anderlecht, Belgium; 16 Department of Endocrinology, UCL Cliniques Universitaires Saint-Luc, Brussels, Belgium; 17 Department of Endocrinology and Metabolism, Copenhagen University Hospital, Rigshospitalet, Copenhagen, Denmark; 18 Department of Endocrinology, Erasmus MC: University Medical Center Rotterdam, Rotterdam, Netherlands; 19 Department of Endocrinology/Medicine, Fundacio de Gestio Sanitaria Hospital de la Santa Creu i Sant Pau, Barcelona, Spain; 20 Department of Paediatric Endocrinology; Great, Ormond Street Hospital -NHS Foundation Trust, London, UK; 21 Department of Endocrinology, Haukeland University Hospital, Bergen, Norway; 22 Department of Endocrinology, Institute of Endocrinology, Lithuanian University of Health Sciences, Kaunas, Lithuania; 23 Hospital Universitario Cruces /Biocruces Bizkaia / EHU / CIBERDEM-CIBERER, Valencia, Spain; 24 Department of Endocrinology, IRCCS Ospedale Policlinico San Martino, Genova, Italy; 25 Department of Endocrine and Metabolic Diseases, IRCCS Istituto Auxologico Italiano and BIOMETRA, University of Milan, Milan, Italy; 26 Department of Endocrinology, Department of Molecular Medicine and Surgery, Karolinska University Hospital, Karolinska Institute, Stockholm, Sweden; 27 Department of Endocrinology and Department of Thrombosis and Hemostasis, Leiden University Medical Center, Leiden, Netherlands; 28 Department of Medicine IV, Ludwig-Maximilian-University Munich, Munich, Germany; 29 Department of Endocrinology and Metabolism, Medical University of Vienna, Center for Rare Endocrinologic Diseases, Vienna, Austria; 30 Department of Endocrinology, NHS Greater Glasgow and Clyde Board, Glasgow, UK; 31 Department of Endocrinology, Morbid Obesity and Preventive Medicine, Oslo University Hospital HF, Oslo, Norway; 32 Department of Pediatrics, Endocrine Unit, Scientific Institute San Raffaele, Milan, Italy; 33 Department of Endocrinology, Radboud University Nijmegen Medical Centre -Including Amalia’s Children Hospital, Nijmegen, Netherlands; 34 Institute of Medicine, Sahlgrenska Academy, University of Gothenburg, Gothenburg, Sweden; 35 Department of Endocrinology, Sahlgrenska University Hospital, Gothenburg, Sweden; 36 Department of Internal Medicine and Oncology, Semmelweis University, Budapest, Hungary; 37 Department of Endocrinology, Tartu University Hospital, Tartu, Estonia; 38 Department of Molecular Genetics, Function and Therapy, The Cyprus Institute of Neurology and Genetics, Nicosia, Cyprus; 39 Department of Pediatrics, Pediatric Endocrinology Clinic, Aretaeio Hospital Nicosia, Nicosia, Cyprus; 40 Department of Experimental and Clinical Biomedical Sciences, University of Florence, Florence, Italy; 41 Department of Pediatrics, University Hospital Martin, Jessenius Medical Faculty Comenius University, Martin, Slovakia; 42 Department of Endocrinology, University Hospital Southampton -NHS Foundation Trust, Southampton, UK; 43 Department of Endocrinology, University Hospital Würzburg, Würzburg, Germany; 44 Centre for Endocrinology, Diabetes and Metabolism, Birmingham Health Partners, Birmingham, UK; 45 Department of Endocrinology, University Medical Centre Groningen, Groningen, Netherlands; 46 Department of Endocrinology, Diabetes and Metabolic Diseases, University Medical Centre Ljubljana, University Children’s Hospital, Ljubljana, Slovenia; 47 Department of Endocrinology, Medical University Sofia, USHATE “Acad. Ivan Penchev”, Sofia, Bulgaria; 48 Department of Endocrinology, UZ Brussels, Jette, Belgium

**Keywords:** Cushing’s syndrome, Hypercortisolism, Hemostasis, Venous thromboembolism, Thromboprophylaxis, Guidelines, Endo-ERN survey

## Abstract

**Background:**

Cushing’s syndrome (CS) is associated with an hypercoagulable state and an increased risk of venous thromboembolism (VTE). Evidence-based guidelines on thromboprophylaxis strategies in patients with CS are currently lacking. We aimed to map the current clinical practice for thromboprophylaxis management in patients with CS across reference centers (RCs) of the European Reference Network on Rare Endocrine Conditions (Endo-ERN), which are endorsed specifically for the diagnosis and treatment of CS. Using the EU survey tool, a primary screening survey, and subsequently a secondary, more in-depth survey were developed.

**Results:**

The majority of the RCs provided thromboprophylaxis to patients with CS (n = 23/25), although only one center had a standardized thromboprophylaxis protocol (n = 1/23). RCs most frequently started thromboprophylaxis from CS diagnosis onwards (n = 11/23), and the majority stopped thromboprophylaxis based on individual patient characteristics, rather than standardized treatment duration (n = 15/23). Factors influencing the initiation of thromboprophylaxis were ‘medical history of VTE’ (n = 15/23) and ‘severity of hypercortisolism’ (n = 15/23). Low-Molecular-Weight-Heparin was selected as the first-choice anticoagulant drug for thromboprophylaxis by all RCs (n = 23/23). Postoperatively, the majority of RCs reported ‘severe immobilization’ as an indication to start thromboprophylaxis in patients with CS (n = 15/25). Most RCs (n = 19/25) did not provide standardized testing for variables of hemostasis in the postoperative care of CS. Furthermore, the majority of the RCs provided preoperative medical treatment to patients with CS (n = 23/25). About half of these RCs (n = 12/23) took a previous VTE into account when starting preoperative medical treatment, and about two-thirds (n = 15/23) included ‘reduction of VTE risk’ as a goal of treatment.

**Conclusions:**

There is a large practice variation regarding thromboprophylaxis management and perioperative medical treatment in patients with CS, even in Endo-ERN RCs. Randomized controlled trials are needed to establish the optimal prophylactic anticoagulant regimen, carefully balancing the increased risk of (perioperative) bleeding, and the presence of additional risk factors for thrombosis.

**Supplementary Information:**

The online version contains supplementary material available at 10.1186/s13023-022-02320-x.

## Background

Cushing’s syndrome (CS) is characterized by excessive tissue exposure to glucocorticoids, caused by either exogenous administration of synthetic glucocorticoids, or excessive endogenous secretion of cortisol. Endogenous CS is rare, with an estimated incidence of 0.2–5.0 cases per million inhabitants per year in various populations, whereas its prevalence is close to 39–79 cases per million inhabitants [1]. Endogenous CS is most commonly caused by a pituitary corticotroph adenoma (Cushing’s Disease, CD), accounting for 70% of all CS cases, and least frequently by adrenocorticotropic hormone (ACTH)-secreting non-pituitary tumors (ectopic ACTH and corticotropin-releasing hormone syndrome, CRH). ACTH-independent CS, is most commonly caused by an unilateral adrenal adenoma, or in fewer cases by bilateral micronodular, or macronodular adrenal hyperplasia, or adrenal carcinoma [1].

In recent years, the association between CS and hypercoagulability has gained growing interest. Multiple cohort studies reported an increased risk for venous thromboembolism (VTE), which encompasses pulmonary embolism (PE) and deep vein thrombosis (DVT), in patients with CS, both during the active phase of disease, and in the postoperative period after transsphenoidal surgery or adrenalectomy, and even after biochemical remission [2]. In their systematic meta- analysis, Wagner et al. found an almost 18-fold higher incidence of VTE in patients with CS compared with the general population [3]. A national multicenter cohort study by Stuijver et al. [4] showed an incidence rate of VTE in CS of 14.6 per 1000 person-years, whereas the risk for postoperative VTE in patients with ACTH- dependent CS was 3.4%.

The underlying mechanisms of, and contributing factors for the hypercoagulable state in patients with CS are still under investigation, with observed/reported coagulation profiles in patients with CS being heterogeneously affected. The hemostatic abnormalities most consistently reported in the various studies include increased levels of procoagulant factors, e.g. von Willebrand Factor (vWF), and factor VIII, and increased levels of fibrinolytic inhibitors, e.g. plasminogen activator inhibitor-1 (PAI-1), thrombin activatable fibrinolysis inhibitor (TAFI), and alpha 2-antiplasmin. The currently available reports did not find a correlation between the severity of hypercortisolism and hemostatic abnormalities [2, 3].

However, to date, there have been no prospective studies that have evaluated the effects of prophylactic anticoagulation on the occurrence of VTE in patients with CS, and consequently, evidence-based guidelines on thromboprophylaxis strategies in patients with CS are lacking [5]. Only retrospective series showing a decrease in VTE associated mortality and morbidity after the introduction of postoperative antithrombotic prophylaxis with unfractionated heparin followed by warfarin [6], low-molecular weight heparin with or without mechanical interventions [7], or aspirin [8] have been reported. We, therefore, anticipated and hypothesized that European Reference Centers (RCs) applied various thromboprophylaxis strategies for patients with CS. Using the EU survey tool, a primary screening survey, and subsequently a secondary, more in-depth survey were developed and sent to RCs of the European Reference Network on Rare Endocrine Conditions (Endo-ERN), which are endorsed specifically for the diagnosis and treatment of CS, thus allowing mapping of the current clinical practice for thromboprophylaxis management in patients with CS.

## Results

### Response rates

**Fig. 1:**
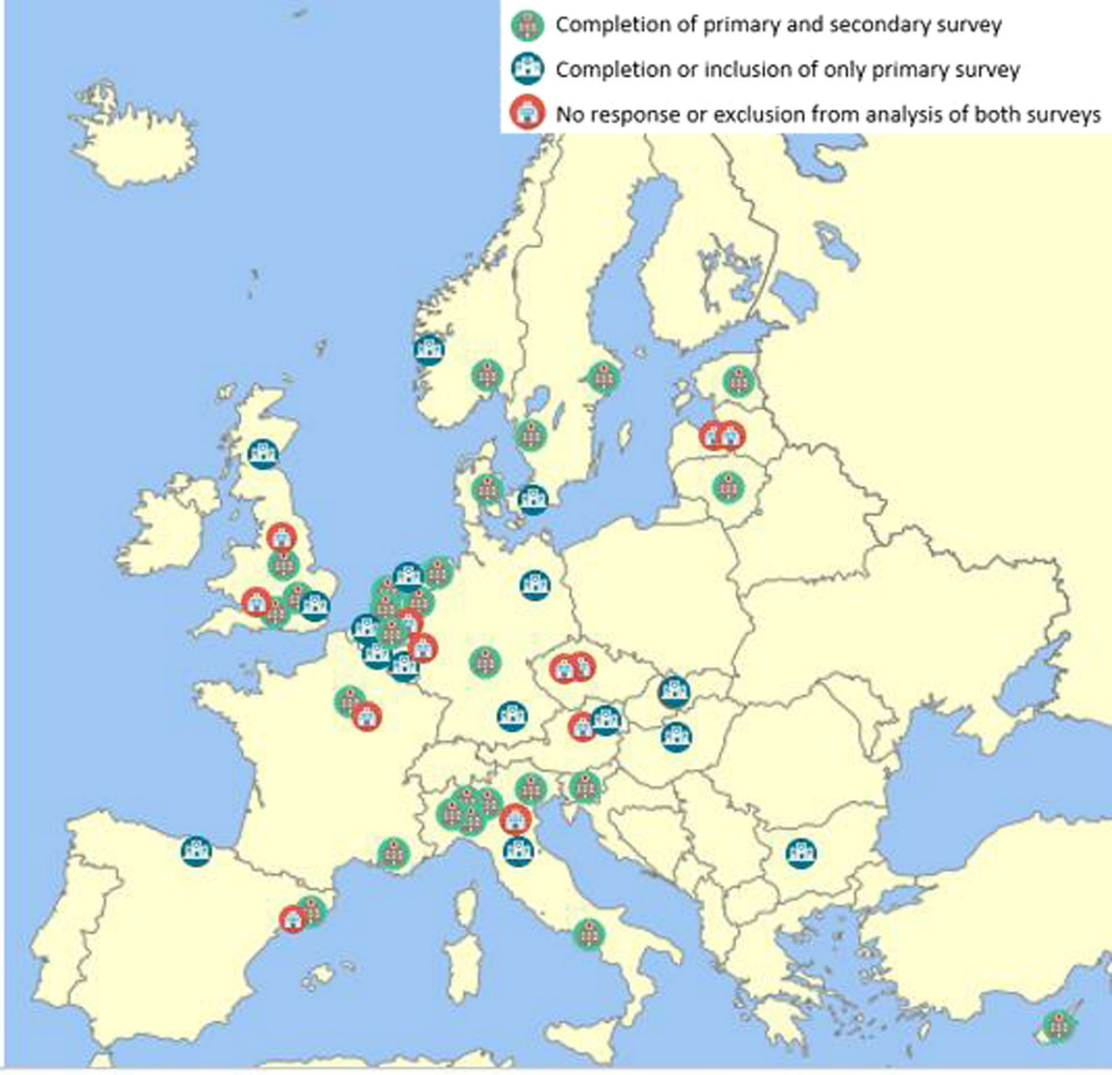
European Landscape of RCs participating in MTG Pituitary and/or MTG Adrenal of Endo-ERN and responder status. Completion of both primary and secondary survey (green icons). Completion of only the primary survey or was included for analysis of only the primary survey (blue icons). Non-responder to the surveys or exclusion from analysis of both surveys (red icons). *Endo-ERN* The European Reference Network on Rare Endocrine Conditions, *MTG* main thematic group, *RC* reference center

Forty-three out of 54 RCs completed the primary survey, of which one RC was excluded because the RC did not treat patients with CS resulting in a final response rate of 78% (n = 42). The secondary survey was sent to the 42 responding RCs of the primary survey, and was completed by 27 RCs of which one RC was excluded due to the lack of both new and chronic patients in their center in the past 2 years. This resulted in a response rate of 62% (n = 26). One response was partial (up to and including the section ‘Treatment of CS’, see Additional file 5). Figure 1 shows an overview of the geographical distribution of RCs per country. Notably, no information on the Cushing population and available treatment modalities due to non-response or exclusion from analysis of both surveys was available for The Czech Republic and Latvia. Slovakia was included for analysis of only the primary survey, and thus, information was partly available.

### Primary survey

The results of the primary survey are summarized in Table 1. The majority of the RCs reported to treat patients with CD (n = 40/42), and benign adrenal CS (n = 39/42). More than half of the RCs (n = 27/42) reported treating the entire spectrum of CS at their center including benign adrenal CS, malignant adrenal CS, CD, and ectopic CS. These RCs were heterogeneously spread across Europe. Additionally, the majority of the RCs (n = 36/42) provided all treatment modalities regarding CS, including surgery, medical treatment, and radiotherapy and administered combination therapy (i.e. combination of surgery and ≥ 1 of the other treatment modalities). The geographical distribution of the RCs, that provided all treatment modalities for patients with CS, showed almost complete coverage of the countries with the exception of Slovakia and Cyprus that have no RC providing all treatment modalities. An overview of the RC’s countries that treated the whole spectrum of CS and provided all treatment modalities is shown in Figs. 4A and 4B included in an supplemental file (see Additional file 1).

**Table 1:** Results of the primary survey

Characteristics	Total number of RCs (N = 42)
*Etiology of CS treated at RC*^a^	
Benign adrenal CS	39 (93%)
Malignant adrenal CS	31 (74%)
Cushing’s disease	40 (95%)
Ectopic CS	33 (79%)
Whole spectrum of CS (i.e. benign adrenal CS, malignant adrenal CS, CD and ectopic CS) treated at RC	27 (64%)
*Treatment modalities for CS available at RC*	
Surgery + medical treatment	3 (7%)
Surgery + medical treatment + combination therapy^b^	2 (5%)
Surgery + medical treatment + combination therapy^b^ + radiotherapy	36 (86%)
Combination therapy^b^	1 (2%)
Preoperative medical treatment routinely provided at RC, yes (%)	16 (38%)
Thromboprophylaxis routinely provided at RC, yes (%)	31 (74%)
*If yes, setting*^a^	
In the inpatient setting	25/31 (81%)
In the ambulatory setting	6/31 (19%)
Presence of a thromboprophylaxis protocol for patients with CS, yes (%)	11 (26%)
Registration of bleeding complication, yes (%)	18 (43%)
Documentation of severity and outcome of bleeding, yes (%)	22 (52%)
Registration of TE events, yes (%)	24 (57%)
*If yes, specific registration of*	
PE + DVT	7/24 (29%)
PE + DVT + AT	17/24 (71%)

*AT* arterial thrombosis, *CS* Cushing’s syndrome, *CD* Cushing’s disease, *DVT* deep vein thrombosis, *PE* pulmonary embolism, *RC* reference center, *TE* thromboembolic

^a^Not mutually exclusive

^b^Combination therapy was defined as combination of surgery and ≥ 1 of the other treatment modalities

Sixteen of 42 RCs routinely provided preoperative medical treatment, and nearly three-quarters of RCs (n = 31) routinely provided thromboprophylaxis to patients with CS, of which the majority (n = 25) gave thromboprophylaxis only in the inpatient setting, while six RCs also prescribed thromboprophylaxis in the ambulatory setting. Eleven of 42 RCs reported to have a dedicated thromboprophylaxis protocol/policy available at their center. Twenty-four of 42 RCs systematically registered TE events, of which the majority (n = 17) specifically registered PE, DVT, and arterial thrombosis (AT), while seven RCs only registered PE, and DVT specifically. Eighteen RCs systematically registered bleeding complications, and twenty-two RCs documented the severity and outcome of the bleeding.

### Secondary survey

#### Definitions

The section on definitions was completed by 26 RCs. First, the definitions of new and chronic patients being used by RCs varied greatly. The majority of the RCs used the following definitions: (a) new patients were defined as patients not previously seen by their center (n = 8), or as treatment naive patients, in addition to any patient not previously seen by their center (n = 8), and (b) chronic patients were defined as patients under active treatment (n = 7). An overview of all used definitions of new and chronic patients by the different RCs is presented in Table 4 enclosed in an supplemental file (see Additional file 2).

#### Epidemiology

Twenty-six RCs were included in the analysis for the section on epidemiology. Complete estimated numbers of new and chronic patients under local care, and numbers of performed transsphenoidal surgeries and adrenalectomies in 2019 and 2020 were provided (Table 5; see Additional file 3). Among the participating RCs, the number of new patients with CS ranged from 0 to 45 in 2019, and from 0 to 56 in 2020. The number of patients with CS under chronic care ranged from 1 to 196 in 2019, and from 0 to 215 in 2020. The highest number of both new and chronic patients with CS was reported by France and the Netherlands, respectively. The number of transsphenoidal surgeries that were performed in 2019 and 2020 ranged from 0 to 16, and 0 to 20, respectively. The number of adrenalectomies in 2019 and 2020 ranged from 0 to 21, and 0 to 20, respectively. The highest numbers of performed transsphenoidal surgeries and adrenalectomies were reported by French RCs. Since only the number of CS patients per RC and the number of patients operated on within 1 year were requested in the survey, the number of newly diagnosed patients and patients operated on may not be the same in a single RC due to the fact that patients diagnosed in 1 year, may have had their surgery in another year.

#### Thromboprophylaxis in Cushing’s syndrome

The section on thromboprophylaxis in CS was completed by 25 RCs. Ten RCs answered that thromboprophylaxis was routinely provided to all patients with CS. Thirteen centers provided thromboprophylaxis only in selected and/or severe cases with or without risk factors for venous thromboembolism. Two centers never provided thromboprophylaxis to patients with CS.

#### Treatment duration of thromboprophylaxis

From the twenty-three RCs that provided thromboprophylaxis routinely, or only in selected/severe cases, the majority (n = 11) started thromboprophylaxis from diagnosis onwards. Six centers started thromboprophylaxis on the day of the surgery, or 1 day prior. Four centers started thromboprophylaxis preoperatively, of which three centers provided specifics regarding the moment of thromboprophylaxis initiation; namely at an average of 7, 14 and 18 days preoperatively. Furthermore, three RCs started thromboprophylaxis postoperatively, of which two RCs started at an average of 1 day, and one RC at an average of 3 days postoperatively. Two RCs reported that the start of thromboprophylaxis for patients with CS varied, and depended on presentation. Having started thromboprophylaxis in patients with CS, the time at which thromboprophylaxis was abrogated was standardized in approximately one-third of the RCs (n = 8/23), and individualized in two-thirds (n = 15/23), as shown in Table 2. The standardized discontinuation of thromboprophylaxis varied greatly between the RCs. One out of eight RCs stopped somewhere between 1 week before to 2 weeks after surgery, one RC stopped between 5 and 6 days postoperatively and two RCs between two to 4 weeks postoperatively. Furthermore, three RCs stopped at 1 month postoperatively and one RC at 3 months postoperatively. The individualized discontinuation of thromboprophylaxis, on the other hand, depended most frequently on the mobility (n = 9/15), and to a lesser extent on remission according to normalization of cortisol production (n = 6/15). One RC used crosslinked fibrin (XDP), prothrombin time (PT), aPTT and fibrinogen to make an individualized decision on the duration of thromboprophylaxis. Four out of 15 RCs reported that treatment duration varied according to the status of the patient, improvement of clinical parameters (e.g. hypertension, hyperglycemia and hypercortisolism) and/or current risk factors.

**Table 2:** Time for initiation and time for abrogation of thromboprophylaxis in patients with Cushing’s syndrome

Characteristic	Total number of RCs (N = 23)
*Time for initiation of thrombo-prophylaxis*^a^	
From diagnosis onwards	11 (48%)
X days preoperatively (mean):	4 (17%)
X = 7	1/4 (25%)
X = 14	1/4 (25%)
X = 18	1/4 (25%)
Not specified	1/4 (25%)
Start on the day before/of the surgery	6 (26%)
X days postoperatively (mean):	3 (13%)
X = 1	2/3 (67%)
X = 3	1/3 (33%)
Other: varies, depends on presentation	2 (9%)
*Time for abrogation of thrombo-prophylaxis*	
Standardized	8 (35%)
Stop 1 week before until 2 weeks after surgery	1/8 (13%)
Stop between 4 and 6 days postoperatively	1/8 (13%)
Stop between 2 and 4 weeks postoperatively	2/8 (25%)
Stop at 1 month postoperatively	3/8 (38%)
Stop at 3 months postoperatively	1/8 (13%)
Individualized^a^	15 (65%)
Stop upon achieving remission according to normalization of cortisol production	6/15 (40%)
As soon as the patient is no longer immobile	9/15 (60%)
Based upon hemostatic parameters	1/15 (7%)
Other: Varies, depends on patient status, improvement of clinical parameters and/or risk factors	4/15 (27%)

*RC* reference center

^a^Not mutually exclusive

#### Factors influencing the initiation of thromboprophylaxis

**Fig. 2:**
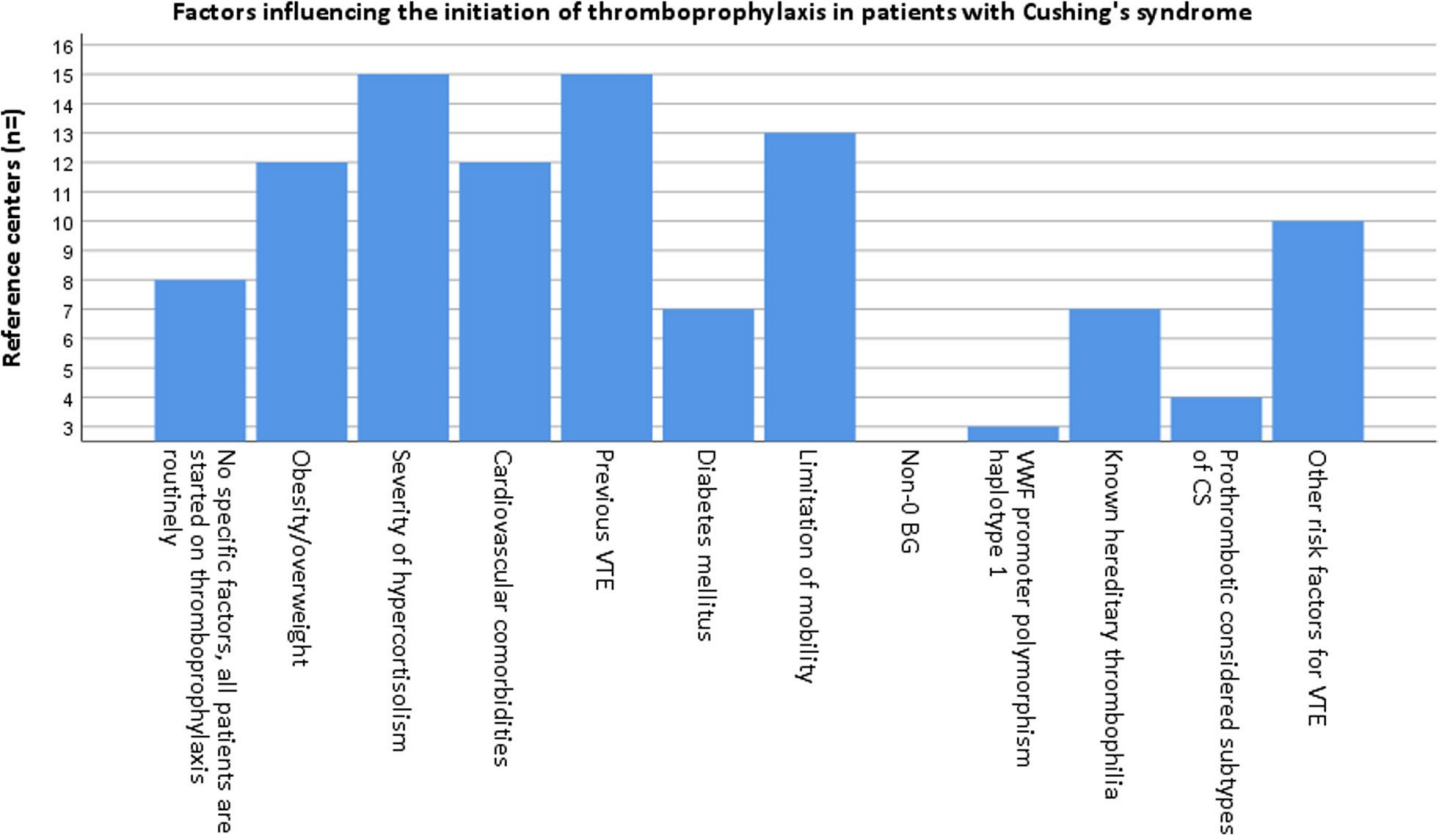
Proportion of responses including each factor influencing initiation of thromboprophylaxis in patients with Cushing’s syndrome (not mutually exclusive). *BG* blood group, *CD* Cushing’s disease, *CS* Cushing’s syndrome, *VTE* venous thromboembolism, *vWF* von Willebrand Factor

The three most frequently selected factors influencing the start of thromboprophylaxis were ‘previous VTE’ (n = 15/23), ‘severity of hypercortisolism’ (n = 15/23), and ‘limitation of mobility’ (n = 13/23), as depicted in Fig. 2. Risk factors for VTE—other than positive history—including older age, cancer and current smoking influenced the start of thromboprophylaxis at ten out of 23 centers. Eight centers started thromboprophylaxis in all patients with CS regardless of the presence of risk factors. Known hereditary thrombophilia (e.g. factor V Leiden/Prothrombin 2021a), and vWF promoter polymorphism haplotype 1 were reported to be used in the decision to start thromboprophylaxis by seven, and three centers, respectively, while non-0 blood group (BG) was not considered by any center. Four centers considered the subtype of CS in the decision of starting thromboprophylaxis (Fig. 2). The prothrombotic considered subtypes of CS most frequently named by these centers were ectopic ACTH/CRH syndrome (n = 3/4) and malignant adrenal CS (n = 3/4), and, to a lesser extent, CD (n = 1/4).

#### Anticoagulant treatment and hereditary screening for thrombophilia in Cushing’s syndrome

All twenty-three RCs that routinely provided thromboprophylaxis, or only in selected/severe cases reported low-molecular-weight-heparin (LMWH) as the first-choice anticoagulant drug for thromboprophylaxis in patients with CS. Direct oral anticoagulants including apixaban, rivaroxaban, dabigatran and edoxaban were not reported. A thromboprophylaxis protocol for patients with CS was provided by only one of 23 centers. All 25 RCs including the centers that never provided thromboprophylaxis answered the question whether they routinely screened for hereditary thrombophilia during diagnostic work up. One RC reported to perform this screening test routinely.

#### Role of venous thromboembolism in preoperative medical treatment of CS

Twenty-five RCs completed the section on preoperative medical treatment in CS. Twenty-three RCs answered that preoperative medical treatment was provided to patients with CS (routinely to all patients or only in selected and/or severe cases). About half of these RCs (n = 12/23) took a previous VTE into account when starting preoperative medical treatment, and about two-thirds (n = 15/23) included ‘reduction of VTE’ as a goal of treatment.

#### Indications for the initiation of postoperative thromboprophylaxis

**Fig. 3:**
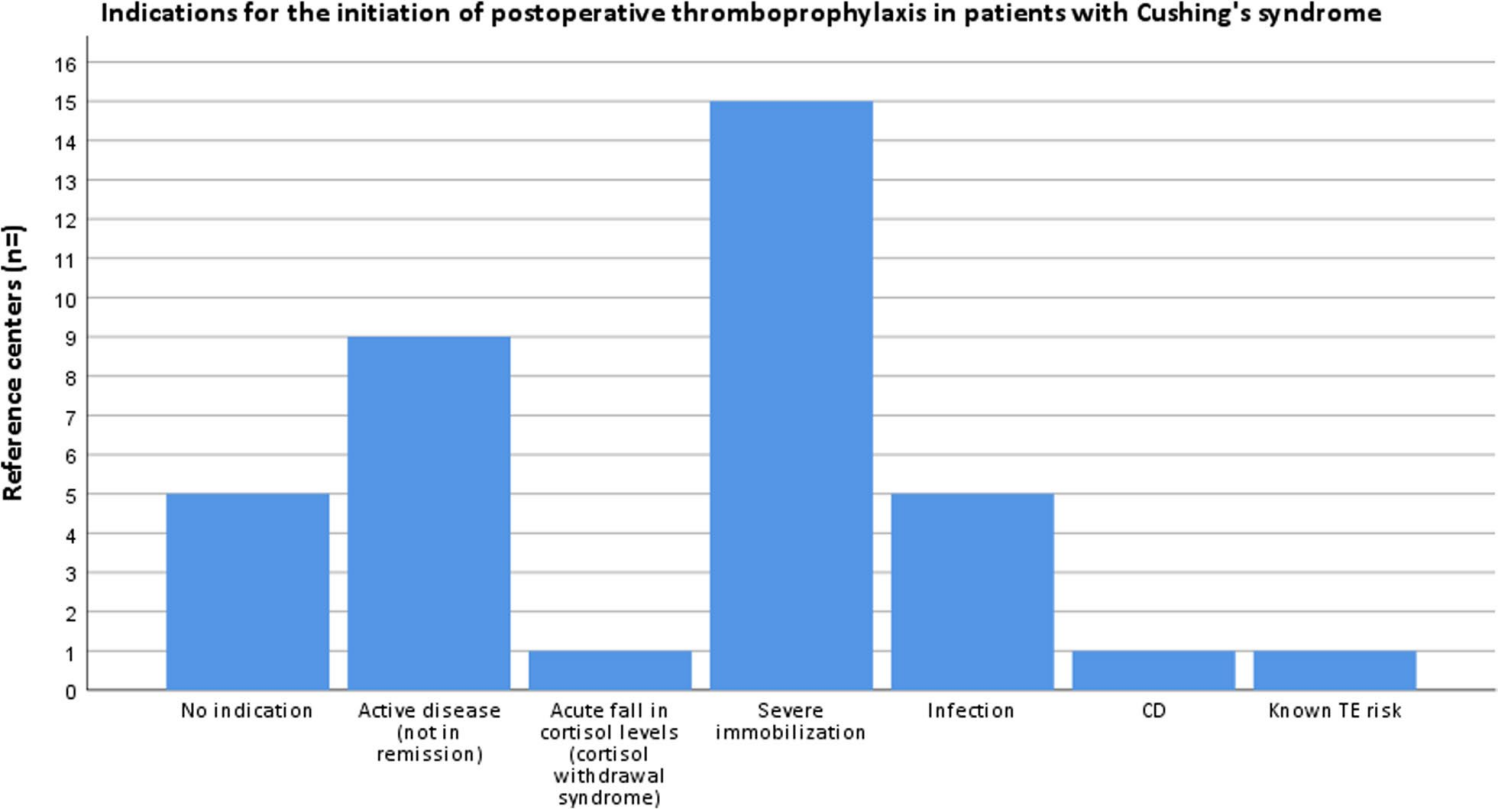
Proportion of responses from each indication for the initiation of postoperative thromboprophylaxis in patients with Cushing’s syndrome (not mutually exclusive). *CD* Cushing’s disease, *TE* thromboembolic

Twenty-five RCs completed the section on postoperative thromboprophylaxis (if not (routinely) provided preoperatively) and follow-up care in CS. Five RCs reported not to routinely prescribe thromboprophylaxis in the postoperative setting (Fig. 3). The most frequently selected indication for postoperative thromboprophylaxis was ‘severe immobilization’ (n = 15/25); ‘known thromboembolic risk’ was reported by one center as shown in Fig. 3.

#### Follow-up care

Six out of 25 centers included hemostatic parameters in routine postoperative laboratory testing. These hemostatic parameters are shown in Table 3. Nine out of 25 centers routinely provided graduated compression stockings to patients with CS after surgery. From this group of RCs the treatment duration was until hospital discharge at five centers and until complete mobilization at one center. The remaining three centers did not specify the treatment duration.

**Table 3:** Characteristics of postoperative care

Characteristic	Total number of RCs
	(N = 25)
*Hemostatic blood testing as standard postoperative care*	
Yes, namely:	6 (24%)
Thrombocytes + INR	1/6 (17%)
Platelet count + aPTT + PT + vWF + AT III + PS +	1/6 (17%)
PC	1/6 (17%)
aPTT + PT	1/6 (17%)
aPTT + INR + D-dimer + fibrinogen	1/6 (17%)
aPTT + PT + INR + D-dimer	1/6 (17%)
aPTT + PT + fibrinogen + XDP	
*Graduated compression stockings as standard postoperative care*	
Yes	9 (36%)

*aPTT* activated partial thromboplastin time, *AT-III* antithrombin III, *PC* protein C, *PS* protein S, *PT* prothrombin time, *RC* reference center, *vWF* von Willebrand Factor, *XDP* serum crosslinked fibrin

## Discussion

This study examined the current clinical practice for thromboprophylaxis management in patients with CS across Endo-ERN RCs. This study provides valuable insight into the large variety of thromboprophylaxis strategies for patients with CS, and the limited availability of protocols on thromboprophylaxis even in the reference centers of Endo-ERN that have been endorsed as expert centers for the diagnosis and treatment of CS.

CS is associated with hypercoagulability and an increased risk of VTE (i.e. PE or DVT) both during the active phase of the disease, in postoperative setting, and even after biochemical remission [2]. There are currently no treatment studies on thromboprophylaxis of CS and no guidelines on the use of thromboprophylaxis for patients with CS, and therefore thromboprophylaxis management is committed to each center’s clinical practice [5].

The in-depth assessment of thromboprophylaxis management showed that the majority of the RCs provided thromboprophylaxis routinely to all patients with CS or only in selected/severe cases (n = 23/25), however, a thromboprophylaxis protocol for patients with CS was unavailable in the vast majority of them (n = 22/23). Thromboprophylaxis was mostly started from diagnosis onwards, whereas the moment of stopping thromboprophylaxis was merely based on individual characteristics rather than standardized treatment duration. Because active CS is associated with a moderate to high risk on VTE [2–4] there is a rationale to start with thromboprophylaxis at diagnosis. On the other hand, treatment with anticoagulation is accompanied by an increased risk of major bleeding, which has been reported to be between 2.8 and 6 per 100 person years [3]. However, the bleeding tendency in CS may be only theoretical, as no increased bleeding complications were found in patients with CS undergoing laparoscopic adrenalectomy [9]. Although CS is associated with bruising and poor wound healing, these manifestations are thought to be the result of alterations in synthesis of skin components rather than specific coagulation disorders [10]. Future studies should assess additional risk factors to determine which patients are particularly at risk for VTE and would benefit from thromboprophylaxis. The individualized decision to abrogate depended mostly on the mobility status of the patient. Risk factors that influenced the initiation of thromboprophylaxis in patients with CS were most frequently reported to be ‘previous VTE’ and ‘severity of hypercortisolism’, and LMWH was selected as the first-choice anticoagulant drug by all RCs. Furthermore, the majority of RCs reported ‘severe immobilization’ as an indication to start postoperative thromboprophylaxis in patients with CS if not (routinely) provided preoperatively, and lastly, did not provide standardized testing for hemostatic parameters in the postoperative care of CS.

A thromboprophylaxis protocol for patients with CS was provided by only one center. This center referred to a recently published article by Barbot et al [11]. In this article, perioperative multidisciplinary management of patients with sellar lesions submitted for transsphenoidal surgery was described and suggested. Specifically for patients with CD, the clinical practice included elastic compression stockings for every patient from the day of admission until full mobilization, treatment with enoxaparin 4000 U once daily, doubling the dose for patients with a body weight above 80 kg for 30 days, starting 24 h after the surgical procedure. However, this protocol did not compromise the whole spectrum of CS [11].

As no studies have been conducted on thromboprophylaxis management in patients with CS, we compared our findings with currently available reports on closely related topics. First, in our study, multiple factors were reported that were taken into account in the decision of thromboprophylaxis initiation in patients with CS. Currently available studies reported multiple risk factors that may be associated with the hypercoagulable state of CS and to our knowledge, no evidence- based VTE risk assessment model for patients with CS has been published thus far [3, 12–14]. In our study, the severity of hypercortisolism was one of the most frequently reported factors that influenced the initiation of thromboprophylaxis. One study found that patients with CS developing VTE had significantly higher plasma cortisol concentrations, compared with CS patients without VTE [12]. However, this was a retrospective study with a very small sample size. Multiple studies found no correlations between the severity of hypercortisolism, and coagulation and fibrinolysis indexes, which was confirmed by Wagner et al. in their recently published systematic meta-analysis [3, 15, 16].

Furthermore, in our study we found a limited role for the measurement of coagulation parameters in the thromboprophylaxis management of CS applied by the Endo-ERN expertise centers. Only one RC reported that the ending of thromboprophylaxis in patients with CS depended on the results of hemostatic variables, including XDP, PT, aPTT and fibrinogen. Additionally, only six RCs reported that hemostatic parameters were screened routinely during follow-up care after transsphenoidal surgery or adrenalectomy. Results of studies examining the hemostatic profiles in patients with CS and the effect of (successful) treatment on these profiles were diverse. A prospective study by Manetti et al. [16] showed an improvement of coagulations indices after successful surgery including vWF, thrombin-antithrombin, antithrombin III, PAI-1, alpha 2-antiplasmin and aPTT. Kastelan et al. [17] found extensive significant improvements of coagulation factors in patients with CS after remission and concluded that the risk of TE 6 months after successful treatment was not greater than the risk faced by healthy individuals. In contrast, a cohort study by Dekkers et al. [13] reported high risks of VTE during the first 3 months following surgery in patients with CS. Furthermore, a study by van der Pas et al. [15] showed no significant changes in aPTT and vWF:Ag in patients with CD after successful pharmaceutical treatment, and additionally showed persistent elevated levels of PAI-1 and alpha 2-antiplasmin. A reason for these contradicting findings may well be the differences in follow-up duration. A systematic meta-analysis by Wagner et al. [3] confirmed the association between CS and VTE, and changes in coagulation parameters including vWF, protein C, protein S, aPTT, fibrinogen and factor VIII, but found no relationship between coagulation parameters and number of thrombotic events. However, more evidence is needed to show whether screening for hemostatic parameters and (changes in) laboratory coagulation metrics can define timing, duration and intensity of (extended) thromboprophylaxis before implementation in daily clinical practice.

In our study we found that four out of 23 centers reported to consider the subtype of CS in the decision of initiation of thromboprophylaxis. The subtypes of CS that were deemed to be associated with an increased risk of TE by these RCs were CD, ectopic ACTH/CRH syndrome and/or malignant adrenal CS. Previous studies showed a higher VTE rate in patients with CD compared to adrenal CS [4, 6]. The reason for the differences in VTE incidence in patients with different etiologies of CS is not clear. Tirosh et al. [18] observed higher AT- III activity and vWF:Ag antigen in patients with CD compared to patients with primary adrenal CS, along with higher baseline mean cortisol levels, and proposed that higher cortisol levels could explain the differences in coagulation profile and increased risk for VTE. However, another study reported no significant differences in coagulation profile between ACTH- dependent and ACTH- independent CS [19]. As to patients with adrenal carcinoma and ectopic ACTH source, the presence of malignancy per se is considered a VTE risk factor, and therefore, these subtypes of CS can be considered prothrombotic in clinical practice, as seen in our study.

The association between preoperative medical treatment and reduction of VTE risk in patients with CS remains controversial. In our detailed assessment of the use of preoperative medical treatment at the different centers, we found that only about half of the responding RCs (n = 12/23) reported to take risk factors for VTE (e.g. older age, cancer and previous VTE) into account in the decision of starting treatment in patients with CS. In addition, about two-thirds (n = 15/23) reported that reduction of the risk of VTE postoperatively was one of the goals of preoperative medical treatment. Preoperative medical treatment might have a role in reducing the likelihood of VTE by reducing the cortisol withdrawal syndrome (i.e. a rapid and large decrease in cortisol exposure after surgery) that can trigger a rebound inflammatory response by withdrawal of the anti-inflammatory effect of cortisol [3]. Stuijver et al. [4] reported a reduced risk ratio of VTE 3 months postoperatively in patients with CS who were medically pretreated before surgery, in comparison to patients who were not. In contrast, a study by Valassi et al., in which data on preoperative medical treatment from The European Registry on Cushing’s syndrome (ERCUSYN) was analyzed, reported no differences in postsurgical morbidities including thromboembolism within 180 days of surgery between patients who received preoperative medical treatment compared to patients who underwent surgery directly. Furthermore, there was little evidence that preoperative medical treatment affected postsurgical outcome [20].

Important limitation of our study is that our findings may be biased due to non-responders and missing data. However, a minimum response rate of 60% was achieved, and the survey questions were mainly independent from each other. We tried to prevent ambiguity in our survey questions by making a clear distinction between start of thromboprophylaxis in an inpatient and/or ambulatory/out-patient setting, and by enquiring about the exact time of initiation of thromboprophylaxis. However, thromboprophylaxis management in general of patients who are not diagnosed with CS or of patients admitted to the RCs for surgery related to a condition other than CS was not surveyed.

## Conclusions

Current clinical thromboprophylaxis management in patients with CS varies considerably across Endo-ERN reference centers. In the absence of prospective studies evaluating thromboprophylaxis on the occurrence of VTE in patients with CS, no evidence-based guidelines on thromboprophylaxis management for patients with CS exist. As the clinical practices have shown to be highly variable, randomized, controlled trials are needed to establish the optimal prophylactic anticoagulant regimen for patients with CS taking into account the increased risk of perioperative bleeding and the presence of additional risk factors for thrombosis.

## Methods

### Aim of the study

The aim of this study was to map the current thromboprophylaxis regimens, (perioperative) treatment practices, and follow-up care after treatment for CS across the (inter)nationally endorsed RCs of the Endo-ERN.

### Study setting

In March 2017, European Reference Networks for rare and complex diseases (ERNs) were installed. ERNs are virtual networks involving RCs across the EU and their primary aim is to enhance cross-border expert consultation and guide conformity for rare and/or complex diseases [21]. The Endo-ERN includes 71 RCs in 19 EU member states. Each of the RCs has been endorsed both nationally and subsequently at the European level for specific expertise for CS, RCs participate in the main thematic disease groups of ‘Adrenal’ and ‘Pituitary’ [22].

### Study design

This was a survey based study, with a primary and secondary survey which are included in Additional files 4 and 5, respectively.

The questionnaires included compulsory questions presented in open-ended and multiple choices and in yes/no-format. The surveys were developed using the EU Survey tool and RCs were approached by email which included a link to the survey. A reminder email was sent approximately 4 weeks after the initial mail-out. RCs that did not respond to the reminder email within 2 weeks after the reminder mail-out were considered non-respondents. Partial completions of the questionnaires were included in the study analysis due to the independent character of the survey questions. The exclusion criteria of the primary survey was the absence of patients with CS, and of the secondary survey was the lack of new and chronic patients with CS in their center in 2019 and 2020. A response rate of 60% was considered sufficient for analysis.

### Study parameters

#### Primary survey

First, a primary survey was developed and send to 54 participating RCs of the Endo-ERN endorsed for the diagnosis and treatment of CS. The primary survey included eighteen questions which served as a screening tool to capture the first essential data for the development of the secondary survey. The questionnaire addressed current practices related to key performance indicators, treatment of CS, and cortisol-lowering treatment prior to surgery, i.e. preoperative medical treatment, prophylactic anticoagulation treatment, and monitoring for thromboembolic events (TE) and bleeding complications in patients with CS.

#### Secondary survey

Next, we developed a secondary survey based on the outcome of the primary survey questionnaire. The secondary survey included 35 questions and was sent to all responders of the primary survey. The section on thromboprophylaxis in CS in the secondary survey was fully completed by RCs that provided thromboprophylaxis to patients with CS. RCs that never provided thromboprophylaxis to patients with CS were requested to answer the questions on ‘hereditary screening for thrombophilia in CS’, ‘indications for the initiation of postoperative thromboprophylaxis’ and ‘follow-up care’. Information on treatment duration of thromboprophylaxis in patients with CS was assessed with questions on the time for initiation of thromboprophylaxis, and the time at which thromboprophylaxis was abrogated. Furthermore, remission of CS was defined as normalization of cortisol production in the survey.

The main goal of the secondary survey was a more in-depth assessment of thromboprophylaxis management in daily clinical practice in patients with CS, protocols for thromboprophylaxis, if any, and (perioperative) treatment practices and follow-up care after transsphenoidal surgery or adrenalectomy in patients with CS. Furthermore, the epidemiological distribution of new and chronic CS patients and performed surgeries were assessed. This was done for both 2019 and 2020 to avoid distortion of information as a result of the COVID-19 pandemic. Lastly, to prevent information bias definitions of new and chronic patients were surveyed too.

### Statistical analyses

Descriptive statistics were used to present data, with categorical variables being presented as number (n), and continuous variables being described as means with ranges. Statistical analysis was performed using SPSS version 25.0.

## Supplementary Information

**Additional file 1:** Characteristics of care for Cushing’s syndrome patients at the reference centers.

**Additional file 2:** Definitions used by the reference centers.

**Additional file 3:** Epidemiological data of Cushing’s syndrome patient population across the Endo-ERN.

**Additional file 4:** Primary survey.

**Additional file 5:** Secondary survey.

## Data Availability

The datasets used and/or analyzed during the current study are available from the corresponding author upon reasonable request.

## Abbreviations

ACTH: Adrenocorticotropic hormone
aPTT: Activated partial thromboplastin time
AT: Arterial thrombosis
CLT: Clot lysis time
CRH: Corticotropin-releasing hormone
CD: Cushing’s disease
CS: Cushing’s syndrome
DVT: Deep vein thrombosis
Endo-ERN: The European Reference Network on Rare Endocrine Conditions
ERN: European Reference Network
LMWH: Low-molecular-weight-heparin
PAI-1: Plasminogen activator inhibitor-1
PE: Pulmonary embolism
PT: Prothrombin time
RC: Reference center
TAFI: Thrombin activatable fibrinolysis inhibitor
TE: Thromboembolic
TSS: Transsphenoidal surgery
VTE: Venous thromboembolism
vWF: Von Willebrand Factor
XDP: Serum crosslinked fibrin

## References

[1] Lacroix A, Feelders RA, Stratakis CA, Nieman LK (2015). Cushing's syndrome. Lancet.

[2] van der Pas R, Leebeek FW, Hofland LJ, de Herder WW, Feelders RA (2013). Hypercoagulability in Cushing's syndrome: prevalence, pathogenesis and treatment. Clin Endocrinol (Oxf).

[3] Wagner J, Langlois F, Lim DST, McCartney S, Fleseriu M (2018). Hypercoagulability and risk of venous thromboembolic events in endogenous Cushing's syndrome: a systematic meta-analysis. Front Endocrinol (Lausanne).

[4] Stuijver DJ, van Zaane B, Feelders RA, Debeij J, Cannegieter SC, Hermus AR (2011). Incidence of venous thromboembolism in patients with Cushing's syndrome: a multicenter cohort study. J Clin Endocrinol Metab.

[5] Koraćević G, Stojanović M, Petrović S, Simić D, Sakač D, Vlajković M (2020). Cushing's syndrome, a risk factor for venous thromboembolism is a candidate for guidelines. Acta Endocrinol (Buchar).

[6] Boscaro M, Sonino N, Scarda A, Barzon L, Fallo F, Sartori MT (2002). Anticoagulant prophylaxis markedly reduces thromboembolic complications in Cushing's syndrome. J Clin Endocrinol Metab.

[7] Barbot M, Daidone V, Zilio M, Albiger N, Mazzai L, Sartori MT (2015). Perioperative thromboprophylaxis in Cushing's disease: what we did and what we are doing?. Pituitary.

[8] Smith TR, Hulou MM, Huang KT, Nery B, de Moura SM, Cote DJ (2015). Complications after transsphenoidal surgery for patients with Cushing's disease and silent corticotroph adenomas. Neurosurg Focus.

[9] Miyazato M, Ishidoya S, Satoh F, Morimoto R, Kaiho Y, Yamada S (2011). Surgical outcomes of laparoscopic adrenalectomy for patients with Cushing's and subclinical Cushing's syndrome: a single center experience. Int Urol Nephrol.

[10] Shibli-Rahhal A, Van Beek M, Schlechte JA (2006). Cushing's syndrome. Clin Dermatol.

[11] Barbot M, Ceccato F, Lizzul L, Daniele A, Zilio M, Gardiman MP (2020). Perioperative multidisciplinary management of endoscopic transsphenoidal surgery for sellar lesions: practical suggestions from the Padova model. Neurosurg Rev.

[12] Zilio M, Mazzai L, Sartori MT, Barbot M, Ceccato F, Daidone V (2016). A venous thromboembolism risk assessment model for patients with Cushing's syndrome. Endocrine.

[13] Dekkers OM, Horváth-Puhó E, Jørgensen JO, Cannegieter SC, Ehrenstein V, Vandenbroucke JP (2013). Multisystem morbidity and mortality in Cushing's syndrome: a cohort study. J Clin Endocrinol Metab.

[14] Koutroumpi S, Daidone V, Sartori MT, Cattini MG, Albiger NM, Occhi G (2013). Venous thromboembolism in patients with Cushing's syndrome: need of a careful investigation of the prothrombotic risk profile. Pituitary.

[15] van der Pas R, de Bruin C, Leebeek FW, de Maat MP, Rijken DC, Pereira AM (2012). The hypercoagulable state in Cushing's disease is associated with increased levels of procoagulant factors and impaired fibrinolysis, but is not reversible after short-term biochemical remission induced by medical therapy. J Clin Endocrinol Metab.

[16] Manetti L, Bogazzi F, Giovannetti C, Raffaelli V, Genovesi M, Pellegrini G (2010). Changes in coagulation indexes and occurrence of venous thromboembolism in patients with Cushing's syndrome: results from a prospective study before and after surgery. Eur J Endocrinol.

[17] Kastelan D, Dusek T, Kraljevic I, Aganovic I (2013). Hypercoagulable state in Cushing's syndrome is reversible following remission. Clin Endocrinol (Oxf).

[18] Tirosh A, Lodish M, Lyssikatos C, Belyavskaya E, Feelders RA, Stratakis CA (2017). Coagulation profile in patients with different etiologies for Cushing syndrome: a prospective observational study. Horm Metab Res.

[19] Kastelan D, Dusek T, Kraljevic I, Polasek O, Giljevic Z, Solak M (2009). Hypercoagulability in Cushing's syndrome: the role of specific haemostatic and fibrinolytic markers. Endocrine.

[20] Valassi E, Franz H, Brue T, Feelders RA, Netea-Maier R, Tsagarakis S (2018). Preoperative medical treatment in Cushing's syndrome: frequency of use and its impact on postoperative assessment: data from ERCUSYN. Eur J Endocrinol.

[21] de Vries F, Bruin M, Cersosimo A, van Beuzekom CN, Ahmed SF, Peeters RP (2020). An overview of clinical activities in Endo-ERN: the need for alignment of future network criteria. Eur J Endocrinol.

[22] Endo-ERN. Overview of specific expertise (MTG). https://endo-ern.eu/specific-expertise/overview-mtg/.

