# Supplementary material for: Current clinical practice for thromboprophylaxis management in patients with Cushing’s syndrome across reference centers of the European Reference Network on Rare Endocrine Conditions (Endo-ERN)
Source: Orphanet J Rare Dis. 2022 May 3;17:178. doi: 10.1186/s13023-022-02320-x (PMC9062860; doi:10.1186/s13023-022-02320-x)
Supplement: Supplementary file 1 — Additional file 1: Characteristics of care for Cushing’s syndrome patients at the reference centers. [file 13023_2022_2320_MOESM1_ESM.docx]

### Supplemental file 1

Title: Characteristics of care for Cushing’s syndrome patients at the reference centers

Description: Overviews of participating reference centers (RC’s) per country that treated the complete spectrum of Cushing’s syndrome (CS) and that provided the complete spectrum of treatment modalities for CS.


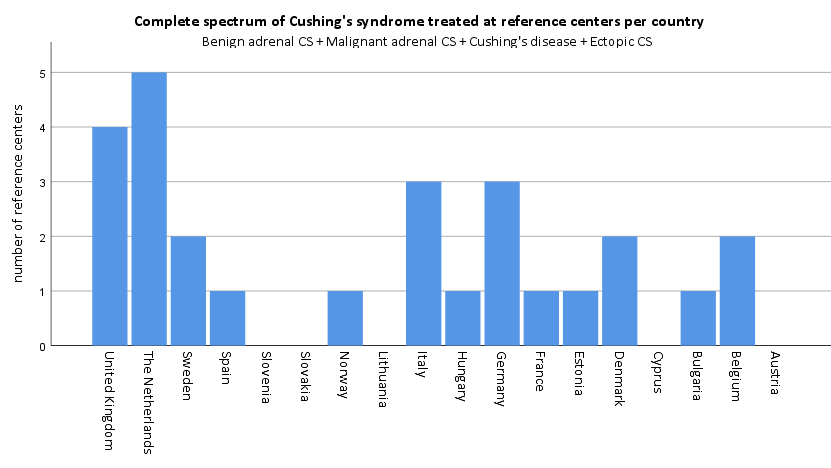


Figure 4A: Overview of the number of reference centers per country that treated the complete spectrum of Cushing’s syndrome (CS).


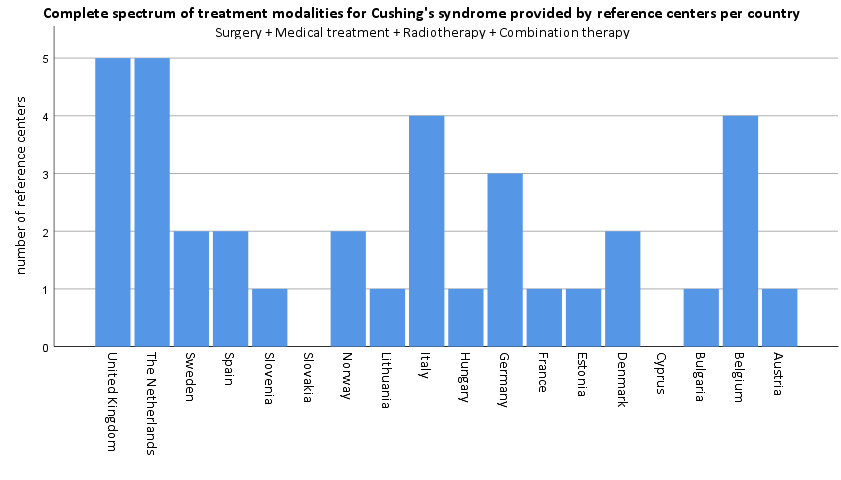


Figure 4B: Overview of the number of reference centers per country that provided the complete spectrum of treatment modalities for CS (n=36). CS, Cushing’s syndrome.
