# Supplementary material for: Current clinical practice for thromboprophylaxis management in patients with Cushing’s syndrome across reference centers of the European Reference Network on Rare Endocrine Conditions (Endo-ERN)
Source: Orphanet J Rare Dis. 2022 May 3;17:178. doi: 10.1186/s13023-022-02320-x (PMC9062860; doi:10.1186/s13023-022-02320-x)
Supplement: Supplementary file 2 — Additional file 2: Definitions used by the reference centers. [file 13023_2022_2320_MOESM2_ESM.docx]

### Supplemental file 2

Title: Definitions used by the reference centers

Description: Overview of used definitions of new patients and patients under chronic care as reported by participating reference centers (RCs).

| Definition(s) of new patients at RC | Total number of RCs (n=26) |
| --- | --- |
| Treatment naive patients | 6 (23%) |
| Patients not previously seen by RC | 8 (31%) |
| Treatment naive patients + Patients not previously seen by RC | 8 (31%) |
| Patients not previously seen by RC + Patients with recurrent disease after initial remission | 1 (4%) |
| Treatment naive patients + Patients not previously seen by RC + Patients with recurrent disease after initial remission | 1 (4%) |
| Treatment naive patients + Patients not previously seen by RC + Patients with recurrent disease after initial remission + Any patient with an exceeding interval between the last and present consultation depending on the Health Record of the RC | 2 (8%) |
| Definition(s) of patients under chronic care at RC | **Total number of RCs (n=26)** |
| Patients under active treatment at RC | 7 (27%) |
| Patients with previous treatment at RC | 4 (15%) |
| Patients with previous treatment currently under affiliated centers referred to RC for a single consultation only, diagnostic tests, or for specific procedure | 2 (8%) |
| Patients under active treatment at RC + Patients with previous treatment at RC | 6 (23%) |
| Patients under active treatment at RC + Patients with previous treatment currently under affiliated centers referred to RC for a single consultation only, diagnostic tests, or for specific procedure | 2 (8%) |
| Patients under active treatment at RC + Patients with previous treatment at RC + Patients with previous treatment currently under affiliated centers referred to RC for a single consultation only, diagnostic tests, or for specific procedure | 5 (19%) |

Table 4: Definition(s) of new patients and of patients under chronic care
