# Supplementary material for: Current clinical practice for thromboprophylaxis management in patients with Cushing’s syndrome across reference centers of the European Reference Network on Rare Endocrine Conditions (Endo-ERN)
Source: Orphanet J Rare Dis. 2022 May 3;17:178. doi: 10.1186/s13023-022-02320-x (PMC9062860; doi:10.1186/s13023-022-02320-x)
Supplement: Supplementary file 3 — Additional file 3: Epidemiological data of Cushing’s syndrome patient population across the Endo-ERN. [file 13023_2022_2320_MOESM3_ESM.docx]

### Supplemental file 3

Title: Epidemiological data of Cushing’s syndrome patient population across the Endo-ERN

Description: Numbers of patients newly diagnosed with CS, patients with CS under chronic care, performed transsphenoidal surgeries (TSSs) and adrenalectomies in 2019 and 2020 at participating reference centers (RCs).

| Name RC | Total new CS patients (n=) | | Total chronic CS patients (n=) | | TSSs (n=) | | Adrenalectomies (n=) | |
| --- | --- | --- | --- | --- | --- | --- | --- | --- |
|  | **2019** | **2020** | **2019** | **2020** | **2019** | **2020** | **2019** | **2020** |
| Aarhus University Hospital | 14 | 13 | 81 | 86 | 7 | 7 | X | X |
| Assistance Publique - Hôpitaux de Marseille | 45 | 56 | 148 | 171 | 15 | 20 | 20 | 20 |
| Assistance Publique-Hopitaux de Paris -Consortium Hôpitaux Cochin, Robert Debré, Necker, St Antoine, La Pitié Salpétrière | 44 | 37 | 92 | 101 | 16 | 10 | 21 | 18 |
| Hospital-University of Padova | 15 | 17 | 168 | 184 | 7 | 5 | 5 | 11 |
| Azienda Ospedaliera Universitaria "Federico II", Napoli | 7 | 1 | 67 | 74 | 3 | 1 | 6 | 2 |
| Azienda Ospedaliero Universitaria Città della Salute e della Scienza di Torino | 5 | 4 | 2 | 1 | 2 | 1 | 3 | 2 |
| UCL Cliniques Universitaires Saint-Luc | 9 | 8 | 77 | 78 | 3 | 4 | 6 | 5 |
| Erasmus MC: University Medical Center Rotterdam | 22 | 17 | 196 | 215 | 7 | 5 | 7 | 6 |
| Fundacio de Gestio Sanitaria Hospital de la Santa Creu i Sant Pau | 11 | 12 | 42 | 43 | 5 | 6 | 3 | 3 |
| Great Ormond Street Hospital - NHS Foundation Trust | 2 | 5 | 4 | 4 | 0 | 3 | 2 | 2 |
| Lithuanian University of Health Sciences | 5 | 7 | 22 | 22 | 3 | 3 | 4 | 3 |
| IRCCS Ospedale Policlinico San Martino – Genova – Italy | 6 | 3 | 24 | 26 | 2 | 2 | 1 | 1 |
| IRCCS Istituto Auxologico Italiano and BIOMETRA | 21 | 18 | 29 | 19 | 2 | 2 | 10 | 10 |
| Karolinska University Hospital | 9 | 7 | 99 | 105 | 4 | 4 | 2 | 2 |
| Leiden University Medical Center | 15 | 14 | 52 | 51 | 15 | 15 | 3 | 2 |
| Oslo University Hospital HF | 11 | 12 | 33 | 36 | 5 | 5 | 5 | 5 |
| Scientific Institute San Raffaele | 2 | 0 | 10 | 10 | 0 | 0 | 1 | 0 |
| Radboud University Nijmegen Medical Centre - including Amalia's children Hospital | 29 | 20 | 113 | 113 | 11 | 10 | 8 | 4 |
| Sahlgrenska University Hospital | 6 | 6 | 84 | 87 | 3 | 5 | 2 | 1 |
| Tartu University Hospital | 4 | 3 | 13 | 12 | 2 | 2 | 1 | 1 |
| The Cyprus Institute of Neurology and Genetics | 1 | 1 | 1 | 0 | 0 | 0 | 0 | 1 |
| University Hospital Southampton - NHS Foundation Trust | 9 | 8 | 26 | 35 | 4 | 5 | 2 | 1 |
| University Hospital Würzburg | X | X | X | X | X | X | X | X |
| University Hospitals Birmingham - NHS Foundation Trust | X | X | X | X | 5 | 5 | X | X |
| University Medical Centre Groningen | 8 | 5 | 73 | 81 | 4 | 2 | 3 | 2 |
| University Medical Centre Ljubljana | 0 | 0 | 1 | 1 | 0 | 0 | 0 | 0 |

Table 5: Numbers of patients newly diagnosed with CS, patients with CS under chronic care, performed TSS and adrenalectomies in 2019 and 2020 at RCs. Missing values are shown as X. CD, Cushing’s disease; CS, Cushing’s syndrome; RC, reference center; TSS, transsphenoidal surgery;
