# Supplementary material for: Current clinical practice for thromboprophylaxis management in patients with Cushing’s syndrome across reference centers of the European Reference Network on Rare Endocrine Conditions (Endo-ERN)
Source: Orphanet J Rare Dis. 2022 May 3;17:178. doi: 10.1186/s13023-022-02320-x (PMC9062860; doi:10.1186/s13023-022-02320-x)
Supplement: Supplementary file 4 — Additional file 4: Primary survey. [file 13023_2022_2320_MOESM4_ESM.docx]

### Supplemental file 4

Title: Primary survey

Description: The primary survey includes 18 questions serving as a screening tool to capture the first essential data for the development of the secondary survey. The questionnaire addressed current practices related to key performance indicators, treatment of Cushing’s syndrome (CS) and pre-treatment prior to surgery, i.e. preoperative medical treatment (PMT), prophylactic anticoagulation treatment, and monitoring for thrombo-embolic (TE) events and bleeding complications in patients with CS.

1. Please select for which Main Thematic Group(s) your HCP participate in? (multiple options possible)
   1. MTG- Pituitary
   2. MTG- Adrenal
2. Would you be interested in participating in studies on the topic of Thromboprophylaxis in patients with Cushing's syndrome? (multiple options possible)
   1. Yes I would like to participate in retrospective chart study
   2. Yes I would like to participate in a RCT
   3. No
3. Does your center have a specific clinical trial unit? (yes/no)
4. Do you see patients with Cushing’s syndrome? (yes/no)
5. Number of new patients per year:
   1. 0-5
   2. 5- 10
   3. 10- 20
   4. >20
6. Total number of patients under chronic care:
   1. 0-10
   2. 10-50
   3. 50-100
   4. >100
7. Please select the appropriate boxes for underlying cause of Cushing Syndrome: (multiple options possible)
   1. Adrenal CS, benign
   2. Adrenal CS, malignant
   3. Cushing’s disease
   4. Ectopic- CS
8. Do you collect clinical data of your patient cohort in a specific database? (yes/no)

If yes: Have (any part of ) these data been published? (yes/no)

If yes, provide reference

1. Are the following treatment modalities provided at your center? (multiple options possible)
   1. Surgery
   2. Medical treatment
   3. Radiotherapy
   4. Combination therapy (e.g. surgery and 1 of the treatment modalities)
2. Please select which medical treatment to you provide treat cortisol excess? (multiple options possible)
   1. Ketoconazole
   2. Metyrapone
   3. Pasireotide
   4. Other

If other, please specify

1. Do you routinely pre-treat prior surgery? (yes/no)

If yes, which medical agent? Please briefly specify protocol regarding duration and dose:

1. Do patients at your center routinely receive thromboprophylaxis? (multiple options possible)
   1. Yes, in the inpatient setting
   2. Yes, in the ambulatory setting
   3. No, only selected and/or severe cases with or without risk factors
   4. No

If yes, please specify:

A, All patients

B. Only severe cases with or without other risk factors

1. Which kind of thromboprophylaxis? (multiple options possible)
   1. Low molecular weight heparin
   2. NOAC
   3. Other

If other, please specify

1. Do you have a specific protocol for thromboprophylaxis? (yes/no)

If yes, please select the specific duration of treatment (multiple options possible)

- 1. From diagnosis onwards
  2. Peri- operatively
  3. 6 weeks after surgery
  4. 12 weeks after surgery
  5. During hospitalization for other reason than elective pituitary or adrenal surgery

1. Do you specifically register bleeding complications? (yes/no)
2. Do you document the severity and outcome of the bleeding complications? (yes/no)
3. Do you specifically register thrombo-embolic events? (yes/no)

If yes, do you register separately (multiple options possible)

- 1. Pulmonary embolism
  2. Deep vein thrombosis
  3. Arterial thrombosis

1. Remarks/comments
