# Supplementary material for: Current clinical practice for thromboprophylaxis management in patients with Cushing’s syndrome across reference centers of the European Reference Network on Rare Endocrine Conditions (Endo-ERN)
Source: Orphanet J Rare Dis. 2022 May 3;17:178. doi: 10.1186/s13023-022-02320-x (PMC9062860; doi:10.1186/s13023-022-02320-x)
Supplement: Supplementary file 5 — Additional file 5: Secondary survey. [file 13023_2022_2320_MOESM5_ESM.docx]

### Supplemental file 5

Title: Secondary survey

Description: The secondary survey includes 35 questions. The main goal of the secondary survey was a more in- depth assessment of thromboprophylaxis (TP) management in daily clinical practice in patients with Cushing’s syndrome (CS), protocols for TP, if any, and (perioperative) treatment practices and follow-up care after transsphenoidal surgery (TSS) or adrenalectomy in patients with CS. Furthermore, the epidemiological distribution of new and chronic CS patients and performed surgeries were assessed for both 2019 and 2020, and definitions of new and chronic patients were surveyed too

***Section 1: Definitions & Epidemiology***

1. How do you define a new patient? (multiple options possible)
2. Treatment naive patients
3. Patients not previously seen by the reference center
4. Any patient with an exceeding interval between the last and present consultation depending on the Health Record of the reference center (for instance more than 12 months)
5. Patients with recurrent disease after initial remission
6. Other, namely:
7. How do you define a patient under chronic care? (multiple options possible)
8. Patients under active treatment at the reference center
9. Patients with previous treatment at the reference center (e.g. patients in complete remission after treatment)
10. Patients with previous treatment currently under affiliated centers referred to the reference center for a single consultation only, diagnostic tests, or for specific procedure
11. Other, namely:
12. Do you have a specific database containing clinical characteristics of patients with Cushing’s syndrome? (yes/no)

- If yes, what kind of database? (for example Excel sheets or files of Electronic Health Record Software)
- If no, are you interested in clinical data collection? (yes/no)

1. What is the number of patients newly diagnosed with the following subtypes of Cushing’s syndrome (CS) in your center in 2019 and 2020?

| CS subtype | Number of new diagnoses (2019) | Number of new diagnoses (2020) |
| --- | --- | --- |
| Cushing’s disease |  |  |
| Ectopic ACTH |  |  |
| Benign adrenal CS |  |  |
| Malignant adrenal CS |  |  |

1. What is the number of patients under chronic care with the following subtypes of Cushing’s syndrome (CS) in your center in 2019 and 2020?

| CS subtype | Number of patients under chronic care (2019) | Number of patients under chronic care (2020) |
| --- | --- | --- |
| Cushing’s disease |  |  |
| Ectopic ACTH/CRH syndrome |  |  |
| Benign adrenal CS |  |  |
| Malignant adrenal CS |  |  |

1. Does discharge of follow- up depend on etiology of Cushing’s syndrome? (yes/no)

- If yes; please specify:

6.2 Is discharge of follow- up related to remission status in patients with Cushing’s syndrome? Please specify (multiple options possible)

1. Discharge upon remission
2. Discharge X months/years in remission

- Please specify:

1. Not related to remission status; lifelong follow- up
2. Not related to remission status; patients are discharged of follow- up when…

- Please specify:

1. What is the frequency of transsphenoidal surgeries for Cushing’s disease (CD) performed in 2019 and 2020?

| Year | Number of transsphenoidal surgeries for CD |
| --- | --- |
| 2019 |  |
| 2020 |  |

1. What is the frequency of adrenalectomies for Cushing’s syndrome (CS) performed in 2019 and 2020?

| Year | Number of adrenalectomies for CS |
| --- | --- |
| 2019 |  |
| 2020 |  |

***Section 2: Treatment of CS***

*The first- line treatment of all forms of Cushing’s syndrome (CS) is surgery. If surgical resection of the primary tumour is not successful or not an option, second- line treatment includes medical treatment. Potential indications for medical therapy of CS include: 1) persistent or recurrent Cushing’s syndrome after transsphenoidal or adrenal surgery; 2) non- feasibility for surgery; 3) acute complications of severe hypercortisolism; 4) pretreatment before surgery.*

*The following questions are about the first-choice medical therapy in the treatment of the different subtypes of CS, and about pretreatment before surgery (i.e. Preoperative Medical Treatment; PMT).*

1. Which medical drug is first-choice in the treatment of Cushing’s disease? (multiple options possible)

- Ketoconazole (x), metyrapone (x), mitotane (x), etomidate (x), osilodrostat (x), levoketoconazole (x), cabergoline (x), pasireotide (x), lanreotide (x), octreotide (x)
- Other, namely:
- Please specify in case of multiple options:

1. Which medical drug is first-choice in the treatment of ectopic ACTH/CRH syndrome? (multiple options possible)

- Ketoconazole (x), metyrapone (x), mitotane (x), etomidate (x), osilodrostat (x), levoketoconazole (x), cabergoline (x), pasireotide (x), lanreotide (x), octreotide (x)
- Other, namely:
- Please specify in case of multiple options:

1. Which medical drug is first-choice in the treatment of benign adrenal CS? (multiple options possible)

- Ketoconazole (x), metyrapone (x), mitotane (x), etomidate (x), osilodrostat (x), levoketoconazole (x), cabergoline (x), pasireotide (x), lanreotide (x), octreotide (x)
- Other, namely:
- Please specify in case of multiple options:

1. Which medical drug is first-choice in the treatment of malignant adrenal CS? (multiple options possible)

- Ketoconazole (x), metyrapone (x), mitotane (x), etomidate (x), osilodrostat (x), levoketoconazole (x), cabergoline (x), pasireotide (x), lanreotide (x), octreotide (x)
- Other, namely:
- Please specify in case of multiple options:

1. Do you provide combination medical therapy (e.g. combination of 2 or more cortisol lowering agents) in patients with severe hypercortisolism?
2. No
3. Yes, routinely
4. Yes, sometimes depending on the case

- If yes, which medical drugs does the first-choice combination therapy consist of?

***Section 3: Preoperative medical treatment (PMT)***

1. In the primary survey you indicated that your reference center provides PMT routinely to patients with Cushing’s syndrome. Is this still applicable? Please specify.
2. Yes, PMT is provided to all patients *Continue with question 16*
3. Only in selected and/or severe cases with or without risk factors *Continue with question 16*
4. No, PMT is never provided *Continue to part 4: Thromboprophylaxis*
5. In the primary survey you indicated that your reference center does not provide PMT routinely to patients with Cushing’s syndrome. Is this still applicable? Please specify.
6. Yes, PMT is never provided *Continue to part 4: Thromboprophylaxis*
7. Only in selected and/or severe cases with or without risk factors *Continue with question 16*
8. No, PMT is provided to all patients *Continue with question 16*
9. Providing PMT (routinely) to patients with Cushing’s syndrome, do you take into account the following factors? (multiple options possible)
10. No specific factors, all patients are medically pretreated before operation
11. Severity of clinical syndrome as reflected by:
12. Difficult-to-treat hypertension
13. Uncontrolled diabetes mellitus or progressive glucose intolerance
14. Biochemical severe cortisol excess
15. Clinical severe syndrome/symptoms
16. Severe psychotic decompensation
17. Other, namely
18. Risk factors for VTE (e.g. older age, cancer, current smoking, previous VTE)
19. Active malignancy with/without treatment
20. What is/are your goals of PMT in patients with Cushing’s syndrome? (multiple options possible)
21. Decrease of cortisol excess
22. Complete normalization of cortisol production
23. Improved regulation of hypertension and/or diabetes mellitus
24. Reduction of VTE risk
25. Prevention of cortisol withdrawal syndrome
26. Reduction of infectious complications
27. Reduction of other surgery- related complications (e.g. bleeding)
28. Reduction of psychopathology
29. Other, namely:
30. Which medical drug is first-choice in the preoperative medical treatment of the following subtypes of Cushing’s syndrome (CS)? (multiple options possible)
31. Pituitary CS: Ketoconazole (x), metyrapone (x), mitotane (x), etomidate (x), osilodrostat (x), levoketoconazole (x), cabergoline (x), pasireotide (x), lanreotide (x), octreotide (x)

- Other, namely:
- Please specify in case of multiple options:

1. Benign adrenal CS: Ketoconazole (x), metyrapone (x), mitotane (x), etomidate (x), osilodrostat (x), levoketoconazole (x), cabergoline (x), pasireotide (x), lanreotide (x), octreotide (x)

- Other, namely:
- Please specify in case of multiple options:

1. Malignant adrenal CS: Ketoconazole (x), metyrapone (x), mitotane (x), etomidate (x), osilodrostat (x), levoketoconazole (x), cabergoline (x), pasireotide (x), lanreotide (x), octreotide (x)

- Other, namely:
- Please specify in case of multiple options:

1. Do you provide combination preoperative medical treatment therapy (e.g. combination of two or more cortisol lowering agents) in Cushing’s syndrome?
2. No
3. Yes, routinely
4. Yes, sometimes depending on the case

- If yes, which medical drugs does the combination therapy usually consist of?

1. When do you start PMT in patients with Cushing’s syndrome?
2. From diagnosis onwards
3. ***X*** days preoperatively
4. X days postoperatively
5. Other, namely:
6. When do you stop PMT in patients with Cushing’s syndrome?
7. **X** days preoperatively
8. **X** days postoperatively
9. Other, namely:

***Section 4: Thromboprophylaxis in CS***

1. In the primary survey you indicated that your reference center provides thromboprophylaxis routinely to patients with Cushing’s syndrome. Is this still applicable? Please specify.
2. Yes, thromboprophylaxis is provided to all patients *Continue with question 25*
3. Only in selected and/or severe cases with or without risk factors *Continue with question 25*
4. No, thromboprophylaxis is never provided *Continue with question 30*
5. In the primary survey you indicated that your reference center provides thromboprophylaxis only in selected and/or severe cases of Cushing’s syndrome with or without risk factors. Is this still applicable?
6. Yes, only in selected and/or severe cases with or without risk factors. *Continue with question 25*
7. No, thromboprophylaxis is provided to all patients *Continue with question 25*
8. No, thromboprophylaxis is never provided *Continue with question 30*
9. In the primary survey you indicated that your reference center does not provide thromboprophylaxis to patients with Cushing’s syndrome. Is this still applicable?
10. Yes, thromboprophylaxis is never provided *Continue with question 30*
11. Only in selected and/or severe cases with or without risk factors *Continue with question 25*
12. No, thromboprophylaxis is provided to all patients *Continue with question 25*
13. Providing thromboprophylaxis routinely or only in selected and/or severe cases, when do you start thromboprophylaxis in patients with Cushing’s syndrome?
14. From diagnosis onwards
15. X days preoperatively
16. Start on the day before/of the surgery
17. X days postoperatively
18. Other, namely:
19. In starting (perioperative) thromboprophylaxis do you take into account the following factors? (multiple options possible)
20. No specific factors, all patients are started on thromboprophylaxis routinely
21. Obesity/overweight
22. Severity of hypercortisolism
23. Cardiovascular comorbidities
24. Previous VTE
25. Diabetes mellitus
26. Limitation of mobility
27. Non- 0 bloodgroup
28. von Willebrand Factor (VWF) promoter polymorphism haplotype 1
29. Known hereditary thrombophilia (e.g. factor V Leiden/Prothrombin 2021a)
30. Subtype of CS

- If yes: Which subtype(s) of CS is/are considered as a prothrombotic factor? (multiple options possible)
- Cushing’s disease
- Ectopic ACTH/CRH syndrome
- Adrenal CS, benign
- Adrenal CS, malignant

1. Other risk factors for VTE (e.g. older age, cancer, current smoking)
2. Which anticoagulant drug is first-choice for (perioperative) thromboprophylaxis in patients with Cushing’s syndrome? (multiple options possible)
3. Low molecular weight heparin
4. Unfractionated heparin via continuous iv infusion
5. Apixaban
6. Edoxaban
7. Rivaroxaban
8. Dabigatran
9. Other, namely:
10. Please specify in case of multiple options:
11. Having started (perioperative) thromboprophylaxis in patients with Cushing’s syndrome, is the treatment duration standardized or individualized?
12. Standardized

- Continuation X days/weeks postoperatively.

1. Individualized (multiple options possible)
2. Stop upon achieving remission according to normalization of cortisol production.
3. As soon as the patient is no longer immobile
4. Based upon hemostatic parameters

→ If yes, which hemostatic parameters?

1. Other, namely:

1. Can you please share the thromboprophylaxis protocol for patients with Cushing’s syndrome (English version)? (yes/no)
2. If thromboprophylaxis is not (routinely) given (perioperatively), is there an indication for starting in the postoperative setting?
3. No indication
4. Active disease (not in remission)
5. Acute fall in cortisol levels (cortisol withdrawal syndrome)
6. Severe immobilization
7. Infection
8. Other, namely:
9. Do you routinely check for hereditary thrombophilia in patients diagnosed with Cushing’s syndrome (for example Factor 5 Leiden, PT2021a)? (yes/no)
10. What is the frequency of clinical follow- up visits after surgery in case of uncomplicated surgery and post-operative course ?
11. What is the testing frequency of cortisol levels after surgery?
12. Does the postoperative laboratory testing include hemostatic parameters? (yes/no)

- If yes; which hemostatic parameters?

1. Do you routinely provide graduated compression stockings to patients with Cushing’s syndrome after surgery? (yes/no)

- If yes, what is the duration of treatment?
- Not specified
- Until hospital discharge
- Continuously for *X* weeks postoperatively
- Other, namely:
